# Lady Windermere Syndrome: Unravelling an Older Lady's Nightmare

**DOI:** 10.7759/cureus.47601

**Published:** 2023-10-24

**Authors:** Aparna Parvathaneni, Sarat Chandra Malempati

**Affiliations:** 1 Internal Medicine, Gandhi Medical College, Hyderabad, IND; 2 Internal Medicine, Saint Vincent Hospital, Worcester, USA

**Keywords:** tree-in-bud nodularity, voluntary cough suppression, mac infection, bronchiectasis, lady windermere syndrome

## Abstract

Infection with nontuberculous mycobacteria (NTM) is an increasingly important cause of pulmonary disease, particularly in immunocompromised patients or those suffering from chronic lung conditions. However, though rare, non-tubercular mycobacterial infection and bronchiectasis may also occur in an immunocompetent patient. This unusual condition is typically seen in middle-aged or elderly white females, with bronchiectasis having a predilection for the middle lobe and lingula. Here, we present a similar case of *Mycobacterium avium* complex (MAC) infection with middle lobe bronchiectasis in an elderly immunocompetent female, recognized as Lady Windermere Syndrome (LWS).

## Introduction

Reich and Johnson coined the term Lady Windermere syndrome in 1992 [1] to describe six elderly immunocompetent women, with no chronic lung disease or significant smoking history, who developed *Mycobacterium avium* complex (MAC) pulmonary infection limited to the right middle lobe or lingula. They speculated that habitual voluntary cough suppression in these women may have led to the poor draining of secretions from the dependent portion of the lingula or its counterpart, the middle lobe. However, there are no larger studies to support this idea. Another hypothesis is that Lady Windermere syndrome could be a connective tissue disorder due to its association with skeletal abnormalities (pectus excavatum, scoliosis, mitral valve prolapse), according to some studies [2-4]. Here, we describe a patient with Lady Windermere syndrome with thin body habitus and a history of voluntary cough suppression but no skeletal abnormalities.

## Case presentation

An 81-year-old lady presented to the emergency department with shortness of breath and fatigue for two years, which has worsened over the last month. For years, she has experienced repeated episodes of cough accompanied by the production of whitish-yellow sputum (each episode lasting six to eight weeks). She was treated with macrolides and beta-lactams for presumed community-acquired pneumonia, initially improving her symptoms with subsequent recurrence. The patient is socially active and has a history of voluntary cough suppression, especially in the presence of others. She reported to have never been a smoker. Of note, she has multiple comorbidities, including coronary artery disease (CAD), paroxysmal atrial fibrillation, osteoporosis, and vascular dementia.

**Figure 1:**
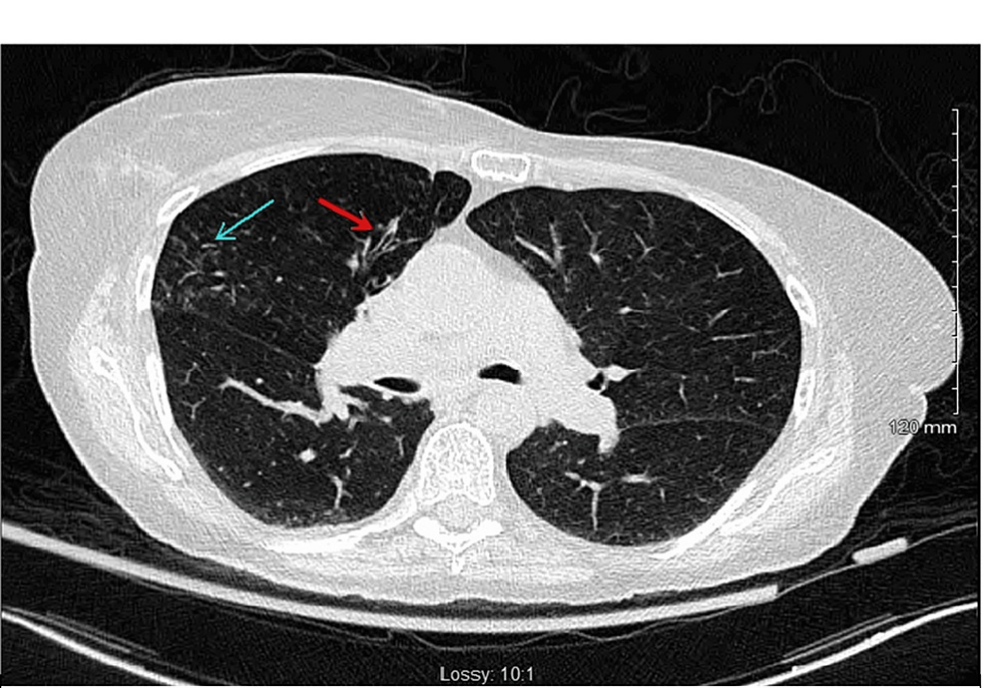
Non-contrast CT imaging of the chest The image is showing bronchiectasis (red arrow) and tree-in-bud nodularity (blue arrow) in the right upper lobe.

**Figure 2:**
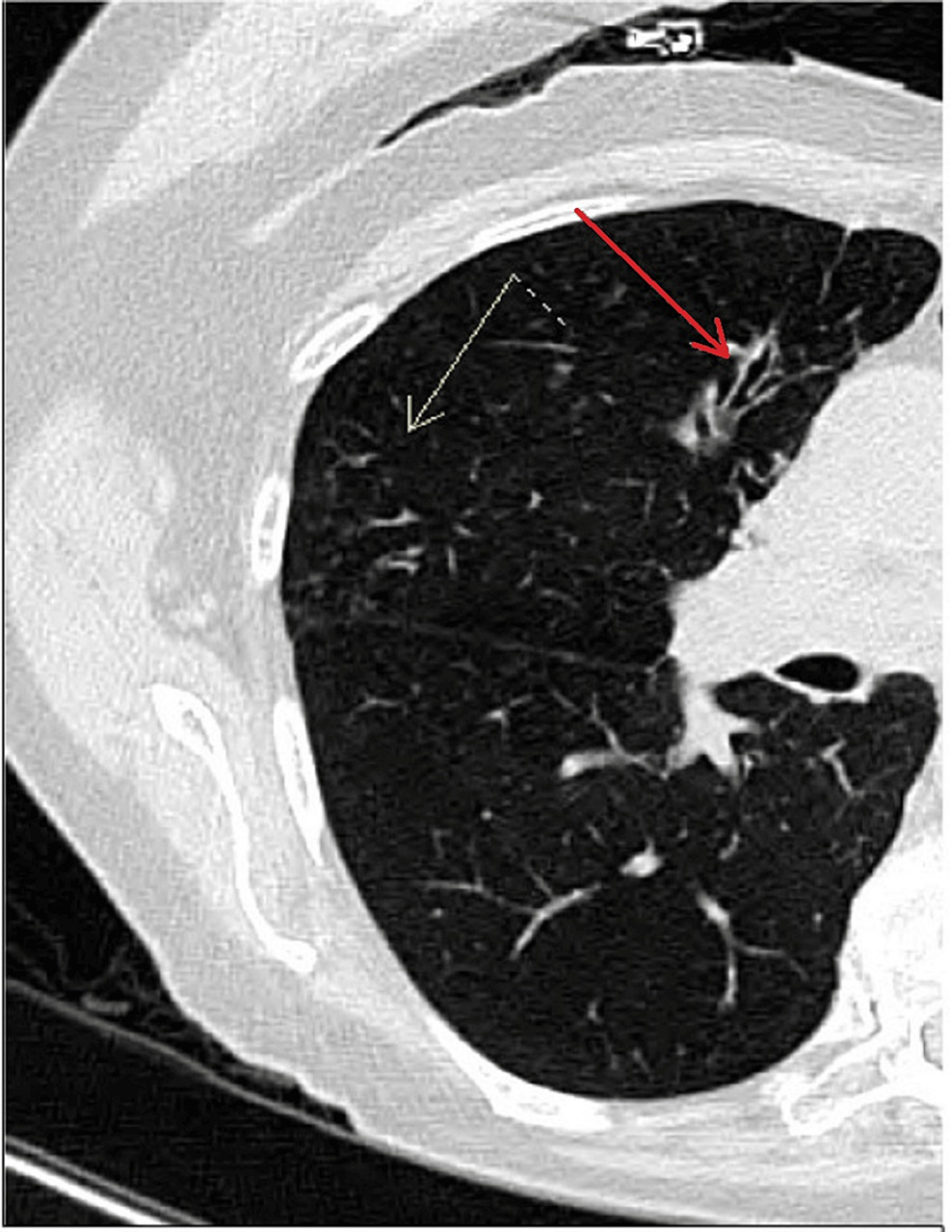
Non-contrast CT image of the chest obtained during a prior visit The image is showing tree-in-bud nodularity (yellow arrow) and bronchiectasis (red arrow) in the right upper lobe.

Physical examination revealed a thin woman with a BMI of 18.1kg/m^2^. She had no chest wall or spinal deformities. Her initial labs, including a complete blood profile, metabolic panel, and renal and liver function tests, were all within normal limits. Her chest X-ray did not reveal any cardiopulmonary processes. However, a chest CT scan revealed tree-in-bud nodularity (Figure 1) in the right middle lobe and associated bronchiectasis, which showed further progression of the disease process as compared to the prior chest CT (Figure 2) two years back.

**Figure 3:**
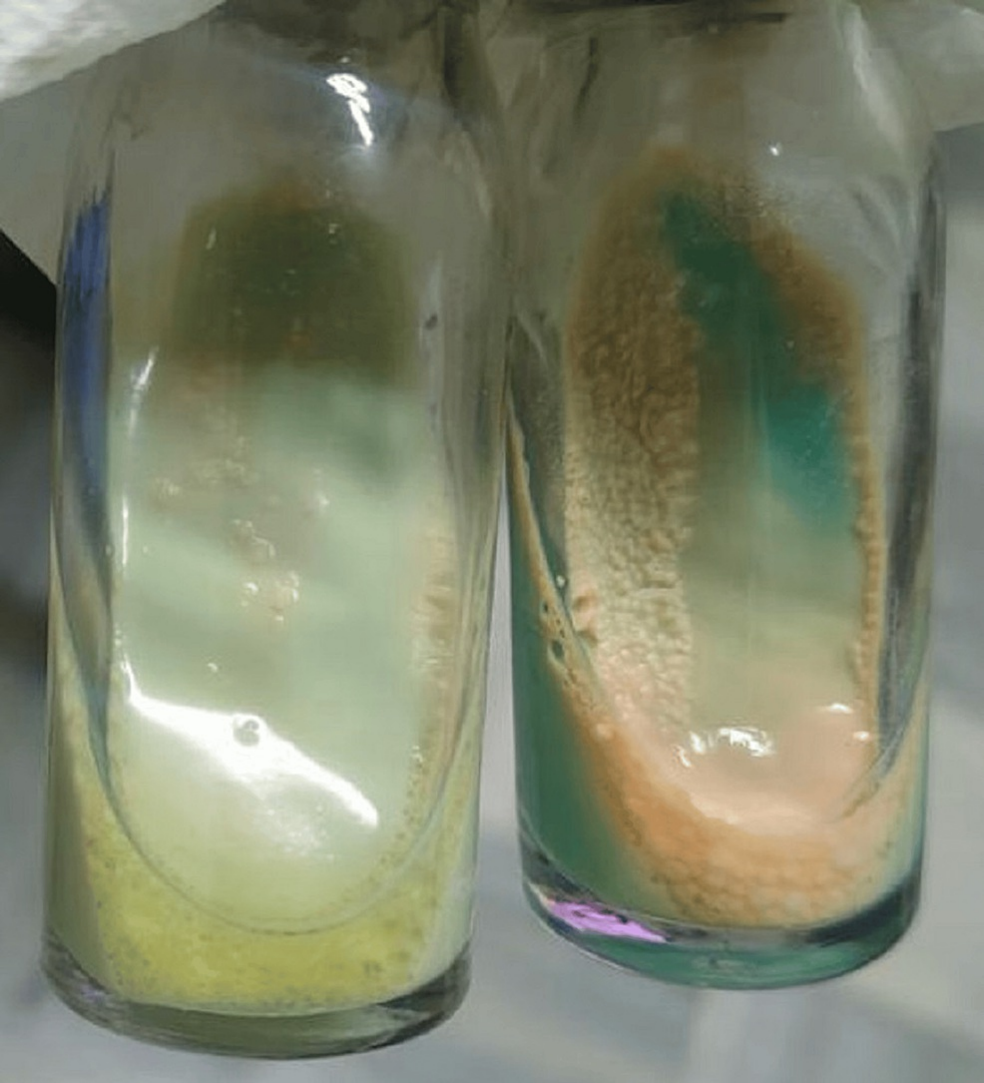
MAC cultures incubated in Löwenstein–Jensen medium in two separate McCartney bottles at 4 weeks. MAC: *Mycobacterium avium* complex

Her QuantiFERON test for tuberculosis and sputum stain for acid-fast bacillus were negative, but cultures eventually revealed MAC (Figure 3).

Given the above findings, a diagnosis of Lady Windermere syndrome was made. Consequently, she was started on a three-drug regimen (rifampicin, ethambutol, and clarithromycin). On follow-up after five months, the patient's respiratory condition was persistently improving without any major side effects.

## Discussion

Non-tubercular mycobacteria are opportunistic pathogens ubiquitously found in the environment [5]. It is interesting to note that although most MAC infections occur in immunocompromised patients or those with pre-existing lung disease, Lady Windermere syndrome/nodular bronchiectasis is a distinct form of MAC infection primarily seen in older women with no apparent immune deficiency or underlying lung condition. While the exact mechanism of pathogenesis for LWS is yet to be confirmed, multiple proposed hypotheses and risk factors exist. It is also unclear whether bronchiectasis precedes or follows *M. avium* infection [6].

Reich and Johnson proposed the hypothesis of voluntary cough suppression [1], which was later observed by Dhillon et al. [7] and Tryfon et al. [8] as well. The predilection of LWS to the middle and lingular lobes can be explained by their narrow, long, and dependent bronchi (in an upright position), with acute angulations predisposing them to infections [7]. In addition to the smaller diameters of the bronchi, a lack of collateral ventilation due to the frequent presence of a complete or partially complete fissure also leads to difficulty in the clearance of secretions without expectoration [9].

Another speculation mentions host anatomic factors, including slender body habitus, low BMI, and skeletal abnormalities. This was described by Iseman et al. [3] and Kim et al. [2], as most of their patients were thin and tall women with a higher incidence of skeletal abnormalities such as scoliosis, pectus excavatum, and mitral valve prolapse. It is possible that these thoracic skeletal abnormalities could contribute to ineffective cough mechanisms and decreased sputum clearance, predisposing them to infection. Due to the frequent occurrence of specific phenotypes associated with LWS, such as skeletal abnormalities and Mitral valve prolapse, some researchers also suggested this might be a connective tissue disorder [3]. Our patient was also a tall female with a low BMI and a history of voluntary cough suppression but no associated skeletal abnormalities or connective tissue disorders.

Chan and Iseman [10] suggested that low estrogen levels could play a role in developing LWS due to its frequent occurrence in postmenopausal women. It has also been observed that decreased subcutaneous fat (low BMI) is linked with an increased adiponectin/leptin ratio, which, in turn, inhibits Th-1 response, accounting for increased susceptibility to *Mycobacterium avium* infection [10].

Diagnosing non-tubercular mycobacterial lung disease can be challenging because of its ubiquitous presence and frequent possibility of sample contamination. According to American Thoracic Society (ATS) guidelines [11], the diagnostic criteria for non-tubercular mycobacterial disease consists of clinical symptoms with compatible radiologic patterns, appropriate exclusion of other criteria, and positive microbiological cultures. Radiographic features include cavitary opacities on chest radiographs or multifocal bronchiectasis with multiple small nodules on high-resolution computed tomography (HRCT). Likewise, our patient had tree-in-bud nodularity on CT in the right middle lobe. Additionally, to rule out contamination, the sputum culture should be positive on at least two separate occasions.

It is important to note that not all patients with pulmonary MAC infection and bronchiectasis need therapeutic intervention. Patients with progressive symptoms or radiographic changes are considered for treatment. With the mainstay of treatment being antibiotics, ATS guidelines [11] recommend a three-drug regimen with a macrolide (clarithromycin or azithromycin) as the primary drug. Additionally, ethambutol and rifampin are added to prevent macrolide resistance. It is a three-times-weekly (intermittent) regimen, with a target of 12 months of negative sputum cultures while on the therapy [11]. Studies conducted by Jeong et al. [12] and Wallace Jr. et al. [13] comparing daily to intermittent therapy showed that patients on intermittent therapy had higher compliance and were less likely to modify their treatment regimen. Additionally, an aminoglycoside can be considered for widespread nodular bronchiectasis or fibrocavitary disease. Further, in patients with failure of antibiotic therapy or symptomatic disease recurrence, surgical intervention in the form of thoracoscopic lobectomy/segmentectomy can be considered [14].

## Conclusions

Lady Windermere syndrome is a rare, poorly understood, isolated right middle lobe or lingular bronchiectasis associated with pulmonary MAC infection predominantly seen in thin, tall females. In immunocompetent patients, pulmonary MAC infections are often overlooked from the radiographic differentials, even when the appearance and clinical picture are characteristics. Non-tubercular mycobacterial lung involvement has been more common in the geriatric populations and is often underdiagnosed, and hence it necessitates higher vigilance and awareness of the clinical symptoms, physical habitus, and associated findings to ensure timely diagnosis and prompt treatment.
